# The impact of merkel cell polyomavirus positivity on prognosis of merkel cell carcinoma: A systematic review and meta-analysis

**DOI:** 10.3389/fonc.2022.1020805

**Published:** 2022-09-30

**Authors:** Aimin Yang, Wilson Adrian Wijaya, Lei Yang, Yinhai He, Ying Cen, Junjie Chen

**Affiliations:** ^1^ Department of Burn and Plastic Surgery, West China Hospital, Sichuan University, Chengdu, China; ^2^ West China School of Stomatology Sichuan University, Chengdu, China

**Keywords:** merkel cell polyomavirus, merkel cell carcinoma, prognosis, systematic review, meta-analysis

## Abstract

**Introduction:**

There are numerous findings over the past decade have indicated that Merkel cell carcinoma (MCC) may have two pathways of pathogenesis: one related to ultraviolet irradiation and the other to the Merkel cell polyomavirus (MCPyV). However, the predictive and clinicopathological value of MCPyV positivity in MCC patients is still debatable. This article aims to examine the most recent data regarding this issue.

**Methods:**

The thorough literature searches were conducted in the Medline Ovid, PubMed, Web of Science, the Cochrane CENTRAL Databases, and Embase Databases until December 31, 2021. The associations between overall survival (OS), Merkel cell carcinoma-specific survival (MSS), recurrence-free survival (RFS), progression-free survival (PFS), clinicopathologic features, and MCPyV positivity were examined in our meta-analysis.

**Results:**

This meta-analysis included a total of 14 studies involving 1595 patients. Our findings demonstrated a significant correlation between MCPyV positivity and improved OS (HR=0.61, 95%CI:0.39-0.94, P=0.026) and improved PFS (HR=0.61, 95% CI: 0.45-0.83, P=0.002). MCPyV positivity did not, however, appear to be associated with either MSS (HR=0.61, 95%CI: 0.28-1.32, P=0.209) or RFS (HR= 0.93, 95%CI: 0.37-2.34, P=0.873). Pooled results revealed a correlation between MCPyV positivity with gender (male vs. female, OR=0.606, 95%CI: 0.449-0.817, P=0.001), histopathological stage (AJCC I-II vs. III-IV, OR=1.636, 95%CI: 1.126-2.378, P=0.010) and primary site (head and neck vs. other sites, OR=0.409, 95%CI: 0.221-0.757, P=0.004).

**Conclusion:**

These results imply that MCPyV positivity may present a promising predictive biomarker for human MCC and call for further study.

## Introduction

Merkel cell carcinoma (MCC), with an incidence of approximately 0.79/100,000, is a rare primary neuroendocrine skin cancer that is more aggressive and has a greater fatality rate than malignant melanoma (1–3). In 1972, the term “trabecular carcinoma of the skin” was first used to characterize the tumor (4). To be noted, the incidence of MCC has been rising yearly in both Europe and the United States since 1995 (3). At present, age, sex, geography, and race are the key factors associated with the incidence (3, 5). The acronym AEIOU summarizes the common features of MCC: asymptomatic, expanding (rapidly) nodules, immunosuppressed, older age, and ultraviolet radiation (UV) exposure. MCC typically manifests as a painless red to violet nodule/nodules on the head and neck or extremities in places exposed to sunlight (5–7).

The association between MCPyV and MCC was firstly confirmed in 2008 when the Cancer Institute of the University of Pittsburgh empirically determined that 80% of MCC specimens were MCPyV positivity (8). MCPyV is almost always present in the skin flora; however, it seldom results in MCC. According to several studies, MCPyV infection is significantly associated with an increased risk of MCC (9). The specific involvement of MCPyV, a naked double-stranded DNA virus of the polyomaviridae, in the development of cancer is unknown. However, it has been reported that persistent expression of one truncated form of the virus large T-antigen (LT) and another small T-antigen (ST) may be associated with tumorigenesis (1, 9).

Clinical and pathological factors are considered independently in the 8th edition of the American Joint Committee on Cancer staging guidelines (10). Tumor size, immune cell infiltration, lymphocytic infiltration, primary tumor site, gender, and nodule growth pattern were among the characteristics linked to prognostic factors (2, 5–7, 10). MCPyV-positive tumors may have a better prognosis, according to some research, whereas others disagree. There has not been a thorough meta-analysis of MCPyV’s impact on clinicopathological parameters and prognosis of MCC. Thereby, we have performed a systematic review and meta-analysis to elucidate the relationship between MCPyV and prognosis of MCC in order to better comprehend this issue.

## Methods

### Literature search

This article complies with the Declaration of Helsinki. Preferred Reporting Items for Systematic Reviews and Meta-analyses (PRISMA) guideline was used to conduct the study. Two authors (YAM and WAW) performed comprehensive searches in the Medline Ovid, PubMed, Web of Science, the Cochrane CENTRAL databases, and Embase from inception to December 31, 2021. The search terms included the following keywords:(“Carcinoma, Merkel Cell” OR “Merkle Tumors” OR “Tumors, Merkle” OR “Merkel Cell Tumor” OR “Tumor, Merkel Cell” OR “Merkel Cell Cancer” OR “Cancer, Merkel Cell” OR “Cell Cancer, Merkel” OR “Merkel Cell Carcinoma”) AND (“Merkel cell polyomavirus” OR “Merkel cell polyomaviruses” OR “polyomavirus, Merkel cell”) AND (“Prognosis” OR “Prognoses” OR “Prognostic Factors” OR “Prognostic Factor” OR “Factor, Prognostic” OR “Factors, Prognostic”). Searches were limited to human participants and English-language publications. The references of the review articles and main researches were also searched in order to avoid omission. Only studies meeting the eligibility criteria outlined below were included in the meta-analysis.

### Eligibility criteria

The extracted data were required to meet the following criteria: (1) the pathological diagnosis of MCC must be confirmed; (2) the presence of MCPyV in MCC tissue was measured by immunohistochemistry (IHC) or polymerase chain reaction(PCR); (3) available data about overall survival (OS), MCC-specific survival (MSS), recurrence-free survival (RFS) and progression-free survival (PFS) that could be accessible; (4) hazard ratio (HR) and 95% confidence interval (CI) of survival data were reported or could be calculated from Kaplan–Meier survival curves;(5) full text available.

Studies that met more than one of the following criteria were excluded: (1) duplicate publications; (2) studies not related to MCPyV and MCC; (3) animal studies, laboratory articles, reviews, letters, meta-analysis, reviews, case reports, or comments; (4) lack of information about survival outcomes or survival curves; (5) insufficient data can be extracted from the article by calculation or by contacting the authors; (6) multiple studies with overlapping samples; (7) The studies with a more significant number of patients were selected when overlapping study samples were identified. Two reviewers(YAM and WAW) independently performed the study selection process, and consensus resolved disagreements.

### Data extraction and quality assessment

Data were extracted by the two independent reviewers(YAM and WAW) using a structured Excel(Microsoft Corp., Redmond, Washington) data collection spreadsheet as a priori. Discrepancies were discussed and resolved within the research team. The following data were retrieved for the included studies: first author, publication year, study design, region, sample material [frozen section(FR) or formalin-fixed paraffin-embedded(FFPE)], number of cases, MCPyV status, detection method of MCPyV presence(PCR primers or immunohistochemistry), patients’ age, gender, tumors’ primary site, stage, size, thickness, angioinvasion, Infiltrating lymphocytes, follow-up time, survival data(OS, MSS, RFS, PFS), HRs. For some studies from which we could not extract HR and CIs directly, Engauge Digitizer software version 12.1 was used to extract survival rate from Kaplan–Meier curves. Two reviewers independently assessed the quality of the eligible studies using the standard Newcastle-Ottawa Scale (NOS) (11). Scores of NOS ≥7 were defined as high quality, 4 to 6 as intermediate quality, and 1 to 3 as low quality. Two reviewers have cross-checked all data, and disagreements were resolved by a third researcher.

### Statistical analysis

This article was performed using Stata version 16.0 (STATA Corp, College Station, TX USA, 2019) for statistical analysis. The correlation between MCPyV positivity and prognosis (OS, MSS, RFS, and PFS) of patients with MCC was evaluated in terms of HRs and 95% CIs. The ORs and 95% CIs were used to evaluate the association between MCPyV positivity and clinicopathological characteristics of MCC. The Q-test result was (I^2^>50% or P<0.05), which indicated heterogeneity between the studies; the random effects model was used for the meta-analysis. Otherwise, a fixed effects model was used. Subgroup analyses were carried out to detect sources of heterogeneity. Begg’s (rank correlation) and Egger’s (regression asymmetry) tests were performed for assessing potential publication bias. Sensitivity analysis was also performed to evaluate the stability of this meta-analysis. The P<0.05 was regarded as statistically significant.

## Results

### Search results and included trials

**Figure 1:**
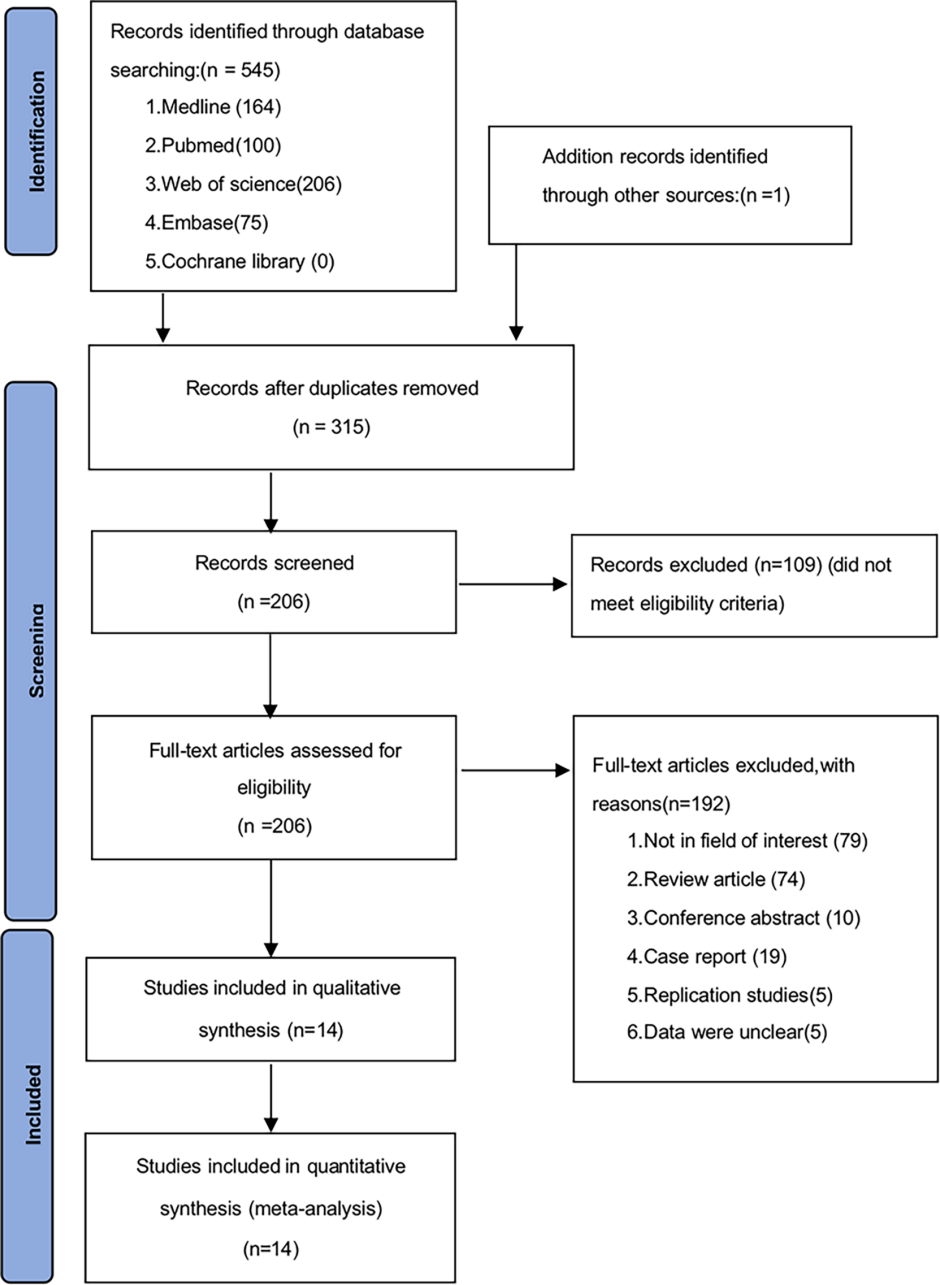
Flow diagram of studies selection.

A total of 546 potentially relevant studies were identified in the literature search. After removing the duplicate articles, 315 articles remained. We then reviewed the titles of the remaining articles as well as their abstracts, and 109 articles were removed. We reviewed each of the remaining 206 articles in full text and finally excluded 192 papers based on the following criteria: 79 studies were not in the fields of interest, 74 studies were review articles, 10 were conference abstracts, 19 were case reports, 5 studies were duplicates, and 5 studies had unclear data. Ultimately, 14 studies (1595 participants) consisting of retrospective cohort studies were included in our meta-analysis. The flowchart and detailed identification of the selection process are shown in **Figure 1**. The main characteristics of the 14 eligible studies published in 10 different countries between 2011 and 2021 are summarized in **Table 1**. The HRs of the included studies and their 95% CIs are summarized in **Table 2**. Thirteen articles had statistics on OS (12–24), 7 studies had data on MSS (12, 15, 17–20, 25), 3 studies had data on RFS (12, 16, 25), and 2 had data on PFS (18, 20). Eleven studies (12–14, 16–18, 20–22, 24, 25) received a score of ≥7 on the NOS score. After quality assessment, all articles were categorized as low risk of bias, although three studies (15, 19, 23) had a moderate risk of bias. The quality of the papers is assessed in **Table 3**.

**Table 1:** Main characteristics of included studies.

First author	Year	Study design	Study region	Patients (n)	Material	MCPyV status	PCR primers	Immunohistochemistry	Age(years)	Gender	Primary site	AJCCClinical stage	Tumor size(cm)	Thickness(mm)	Angioinvasion	Tumour Infiltrating Lymphocytes	Follow-up(months)	Analysis Patients(n)	Outcome	HR Restimate
David Schrama et al. (12)	2011	RC	Europe and Australia	174	NA	Positive: 149	MCPyV	LT	mean73 (66–80)	male:70 female:63 NA:16	Head and neck:41 Extremities:42 Trunk:9 NA:57	I:69 II:39 III:2 NA:39	NA	NA	NA	yes:39 no:55	mean24.9 (4.5–34.1)	Univariate analysis;n=174 Multivariate analysis;n=122	OS,MSS,RFS	paper
						Negative: 25			mean75.1 (68.5–82.5)	male:18 female:5 NA:2	Head and neck:11 Extremities:7 Trunk:2 NA:5	I:11 II:8 III:2 NA:4				yes:7 no:10	mean25.2 (5.2–30.9)			
Brian J. Hall et al. (13)	2012	RC	the United States	36	NA	Positive:17 Negative:19	NA	LT	mean73.7,median77(50-95)	male:15 female:21	Head and neck:19 Extremities:12 Trunk:3 Others:2	NA	NA	NA	NA	NA	mean36.3 (1.8-90.9)	36	OS	paper
Kirsten E Fleming et al. (14)	2014	RC	Canada	83	NA	Positive:16 Negative:21 NA:46	NA	NA	mean75.8 (64.1-87.5)	male:46 female:37	Head and neck:40 Extremities:33 Trunk:7 Others:3	I-II:47 III-IV:28 NA:8	≤2cm:49 >2cm:27 NA:7	≤1mm:43 >1mm:26 NA:14	NA	yes:15 no:65 NA:3	mean40.3 (0-81)	37	OS	indirect
Takeshi Iwasaki et al. (15)	2016	RC	Japan and United Kingdom	41	FFPE	Positive:26	MCPyV	LT	mean71.9 (60.96–82.84)	male:9 female:17	NA	I:10 II:13 III:3	NA	NA	NA	NA	NA	41	OS,MSS	paper
						Negative:15			mean85.0 (75.75-94.25)	male:3 female:12		I:2 II:8 III:2 NA:3								
M. Samimi et al. (16)	2016	RC and PC	France	143	FFPE	Positive: 110 Negative: 33	MCPyV	LT	median78 (31–98)	male:57 female:86	Head and neck:48 Extremities:74 Trunk:15 Others:6	I:49 II:50 III:38 IV:6	NA	NA	NA	NA	median25 (0–148)	OS analysis;n=83 RFS analysis;n=77	OS,RFS	paper
Michiko Matsushita et al. (17)	2017	RC	Japan	41 samples of 35	FFPE	Positive:24	MCPyV	LT	mean74.6(SD ± 9.8)	male:6 female:16	NA	I:9 II:11 III:2	NA	NA	NA	NA	median23 (1-72)	41 samples of 35	OS,MSS	paper
						Negative:17			mean83.3(SD± 9.1)	male:3 female:10		I:2 II:7 III:2								
Ata S Moshiri et al. (18)	2017	RC	the United States	282	NA	Positive: 229	MCPyV	LT4	median71(SD ± 12.6)	male:142 female:87	Head and neck:74 Extremities:100 Trunk:10 Others:45	I:65 II:28 III:61 IV:26	median1.1 (SD ± 1.7)	NA	NA	NA	The 281 persons contributed 1211 person-years	OS/MCC-SS analysis;n=281 PFS analysis;n=247	OS,MSS,PFS	indirect
						Negative: 53			median71(SD ± 11.2)	male:35 female:18	Head and neck:22 Extremities:10 Trunk:6 Others:15	I:10 II:4 III:10 IV:8	median1.9 (SD ± 1.7)							
Lusi Oka Wardhani et al. (19)	2019	RC	Japan and United Kingdom	43	FFPF	Positive:24	NA	NA	mean77.45 (SD ± 10.34)	male:5 female:19	NA	I-II:23 III-IV:1	NA	NA	NA	NA	NA	43	OS,MSS	indirect
						Negative:19			mean84.68 (SD ± 9.63)	male:6 female:13		I-II:15 III-IV:4								
Mai P Hoang et al. (20)	2020	RC	Poland, Taiwan, and the United States	134	NA	Positive:84	NA	LT	≤77:45 >77:39	male:40 female:44	Head and neck:33 Others:51	NA	≤2cm:42 >2cm:42	≤1mm:35 >1mm:47 NA:2	yes:38 no:46	NA	median20 (1–255)	Univariate analysis;n=134 Multivariate analysis;n=133	OS,MSS,PFS	paper
						Negative:50			≤77:21 >77:29	male:34 female:16	Head and neck:32 Others:18		≤2cm:34 >2cm:16	≤1mm:33 >1mm:17	yes:26 no:24					
C Ricci et al. (21)	2020	RC	Italy	95	FFPF	Positive:52 Negative:43	NA	MCPyV	median77(68–84)	male:50 female:45	Head and neck:35 Extremities:43 Trunk:17	I-II:58 III-IV:37	Median2.2 (1.3–3.5)	median11 (6–15)	NA	yes:43 no:39	median24.5(2–132) for died of their disease patient; median17(14–60) for died of other causes patient; median54(5–180) for patients alive at the end of follow-up	82	OS	paper
Hao Xie et al. (22)	2020	RC	the United States	65	FFPF	Positive:39 Negative:26	NA	MCPyV	median73(66–83)	male:44 female:21	NA	I:25 II:10 III:26 IV:2 NA:2	Median1.7 (1.3–2.4)	NA	NA	NA	median23.0 (9.0–47.0)	65	OS	indirect
Hannah Björn Andtback et al. (23)	2021	RC	Sweden	54 in 113	NA	Positive:40 Negative:14	NA	NA	median76(19–100)	male:25 female:29	Head and neck:53 Extremities:44 Trunk:12 Genital area:4	I:64 II:35 III:14	NA	NA	NA	NA	NA	54 female;n=29 male;n=25	OS	indirect
Morgan Guénolé et al. (24)	2021	RC	France	58 in 77	FFPF	Positive:17 Negative:41	NA	MCPyV	median83(49–101)	male:34 female:43	Head and neck:40 Extremities:28 Trunk:9	NA	<2cm:41 >2cm:36	NA	NA	NA	median25.7 (0.7–219.9)	58	OS	paper
Kelly L Harms et al. (25)	2021	RC	the United States	346 samples of 300	FFPF	Positive:177	MCPyV	LT, ISH : TAg	median71.0(SD ± 12.2)	male:91 female:64	Head and neck:43 Extremities:103 Trunk:8 others:1	I:37 II:27 III:83 IV:4	NA	NA	NA	NA	mean40 (range:NA)	MCC-SS analysis;n=173 RFS analysis;n=207	MSS,RFS	indirect
						Negative:151			median78.0(SD ± 19.0)	male:96 female:33	Head and neck:81 Extremities:30 Trunk:12 others:2	I:39 II:7 III:73 IV:5								
						Indeterminate:17 NA:1														

RC, Retrospective cohort; PC, Prospective cohort; FFPE, Formalin-fixed Paraffin-embedded material; PCR, Polymerase Chain Reaction; AJCC, American Joint Committee on Cancer classification; OS, Overall Survival; MSS, MCC-specific Survival; RFS, Recurrence-free Survival; PFS, Progression-free Survival; HR, Hazard Ratio; LT, Large T antigen; MCC, Merkel Cell Carcinoma; MCPyV, Merkel Cell Polyomavirus; SD, Standard Deviation; TAg, Large and Small T antigen; NA, Not Available.

**Table 2:** HRs and their 95% CI of included studies.

First author	Year	HR Restimate	Outcome	HR	95%CI(LL–UL)	p-value
David Schrama (12)	2011	paper	OS	0.750* 1.861**	(0.344–1.636)* (0.519–6.679)**	0.470* 0.341**
			MSS	1.054* 3.664**	(0.362–3.066)* (0.665–20.183)**	0.924* 0.136**
			RFS	1.753* 2.778**	(0.794–3.870)* (0.930–8.298)**	0.165* 0.067**
Brian J. Hall (13)	2012	paper	OS	1.27**	(0.51–3.16)**	0.6067**
Kirsten E Fleming (14)	2014	indirect	OS	0.57*	(0.25–1.33)*	0.197*
Takeshi Iwasaki (15)	2016	paper	OS	0.043* 0.04**	(0.009–0.199)* (0.004–0.386)**	<0.001* 0.005**
			MSS	0.001*	(0.00–26.073)*	0.187*
M. Samimi (16)	2016	paper	OS	0.52* 0.68**	(0.23–1.18)* (0.27–1.72)**	0.12* 0.42**
			RFS	0.52* 0.83**	(0.23–1.15)* (0.38–1.85)**	0.11* 0.65**
Michiko Matsushita (17)	2017	paper	OS	0.101* 0.03**	(0.028–0.370)* (0.004–0.207)**	0.001* < 0.001**
			MSS	0.090* 0.025**	(0.018–0.441)* (0.002–0.346)**	0.003* 0.006**
Ata S Moshiri (18)	2017	indirect	OS	0.76* 0.77**	(0.53–1.08)* (0.51–1.16)**	0.12* 0.21**
			MSS	0.56* 0.67**	(0.36–0.88)* (0.39–1.14)**	0.011* 0.14**
			PFS	0.56* 0.65**	(0.38–0.82)* (0.40–1.04)**	0.003* 0.073**
Lusi Oka Wardhani (19)	2019	indirect	OS	0.24* 0.90**	(0.10-0.64)* (0.16-4.90)**	0.004* 0.898**
			MSS	0.24* 1.53**	(0.05-1.20)* (0.07-32.26)**	0.082* 0.783**
Mai P Hoang (20)	2020	paper	OS	0.52* 1.22**	(0.32–0.83)* (0.49–3.05)**	0.0068* 0.67**
			MSS	0.51* 0.89**	(0.26-0.99)* (0.34-2.30)**	0.046* 0.81**
			PFS	0.72*	(0.42-1.23)*	0.23*
C Ricci (21)	2020	paper	OS	0.290* 0.363**	(0.149–0.564)* (0.161–0.820)**	< 0.001* 0.015**
Hao Xie (22)	2020	indirect	OS	0.30* 0.27**	(0.15-0.63)* (0.09-0.77)**	0.001* 0.02**
Hannah Björn Andtback (23)	2021	indirect	OS	0.77**	(0.38-1.54)**	0.458**
Morgan Guénolé (24)	2021	paper	OS	0.34* 0.69**	(0.16–0.71)* (0.26–1.80)**	0.004* 0.45**
Kelly L Harms (25)	2021	indirect	MSS	0.27* 0.34**	(0.12-0.58)* (0.18-0.65)**	<0.001* 0.001**
			RFS	0.42* 0.47**	(0.25-0.70)* (0.28-0.80)**	<0.001* 0.005**

OS, Overall Survival; MSS, MCC-specific Survival; RFS, Recurrence-free Survival; PFS, Progression-free Survival; HR, Hazard Ratio; CI, Confidence Interval; LL, Lower Limit; UL, Upper Limit; *, Univariate analysis; **, Multivariate analysis.

**Table 3:** Quality assessment *via* Newcastle Ottawa scale and recall bias risk.

Study	Selection				Comparability	Outcome			Total
	①	②	③	④	⑤	⑥	⑦	⑧	
David Schrama et al., 2011 (12)	1	1	1	1	1	1	0	1	7
Brian J. Hall et al., 2012 (13)	1	1	1	1	0	1	1	1	7
Kirsten E Fleming et al., 2014 (14)	1	1	0	1	1	1	1	1	7
Takeshi Iwasaki et al., 2016 (15)	1	1	1	1	1	1	0	0	6
M. Samimi et al., 2016 (16)	1	1	1	1	1	1	1	1	8
Michiko Matsushita et al., 2017 (17)	1	1	1	1	1	1	1	1	8
Ata S Moshiri et al., 2017 (18)	1	1	1	1	1	1	1	1	8
Lusi Oka Wardhani et al., 2019 (19)	1	1	0	1	1	1	0	0	5
Mai P Hoang et al., 2020 (20)	1	1	1	1	1	1	1	1	8
C Ricci et al., 2020 (21)	1	1	1	1	1	1	1	1	8
Hao Xie et al., 2020 (22)	1	1	1	1	1	1	0	1	7
Hannah Björn Andtback et al., 2021 (23)	1	1	0	1	1	1	0	0	5
Morgan Guénolé et al., 2021 (24)	1	1	1	1	1	1	1	1	8
Kelly L Harmset al. 2021 (25)	1	1	1	1	1	1	1	1	8

Note. 1. Representativeness of the exposed cohort; 2. selection of the unexposed cohort; 3.ascertainment of exposure; 4. demonstration that outcome of interest was not present at start of study; 5. comparability of cohorts based on design or analysis; 6. assessment of outcome; 7. was follow-up long enough for outcomes to occur; 8. adequacy of follow up of cohorts.

### Prognostic value of MCPyV positivity for OS in MCC

**Figure 2:**
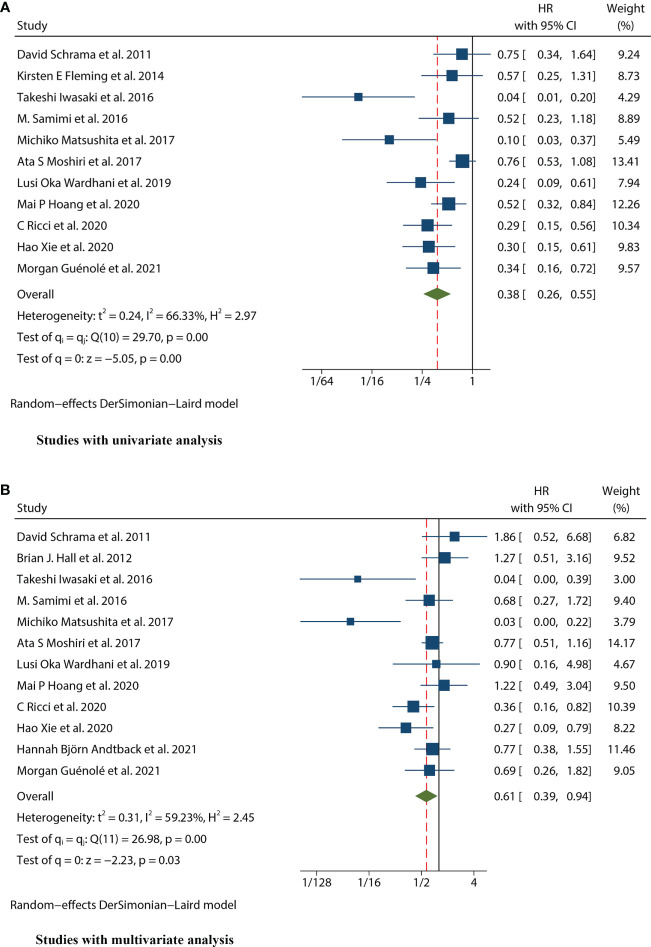
Forest plot of the hazard ratio for the association between the MCPyV and overall survival (OS) in patients with Merkel cell carcinoma. **(A)** univariate analysis. **(B)** multivariate analysis.

**Figure 3:**
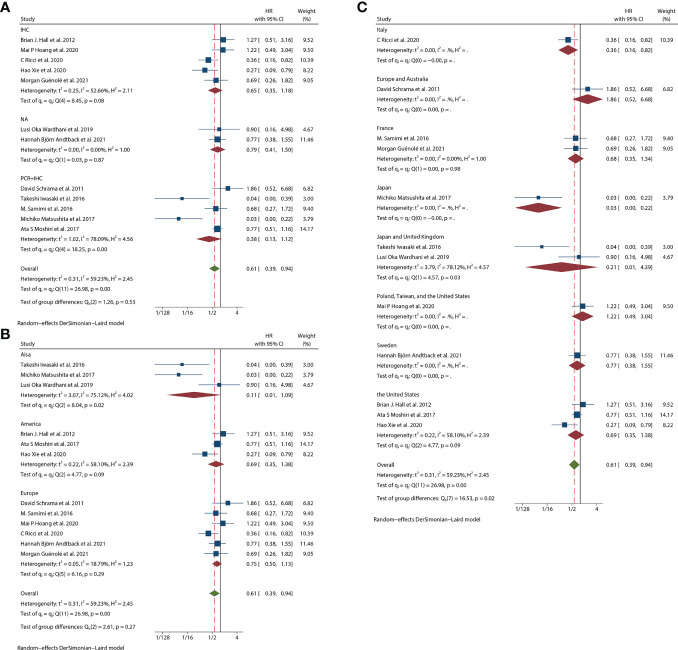
Overall survival (OS) subgroup analyses. **(A)** OS subgroup analysis in term of different detection methods; **(B)** OS subgroup analysis of different continents; **(C)** OS subgroup analysis of different study regions.

Thirteen studies (12–24) consisting of 1249 patients reported OS. Among them, eleven articles (12, 14–22, 24) had univariate analysis statistics on OS, and twelve (12, 13, 15–24) had multivariate. It was found that the MCPyV positivity was a good prognostic indicator for this outcome when analyzing the studies that calculated the combined univariate HR (0.38, 95%CI:0.26–0.55, P=0.000) and multivariate HR (0.61, 95%CI:0.39–0.94, P=0.026) (**Figure 2**). Due to significant heterogeneity (univariate I^2^ = 66.33%, P=0.00 and multivariate I^2^ = 59.23%, P=0.03), we used a random effects model to calculate this meta-analysis. In addition, we refined the subgroup analyses of the detection methods and continents. The results demonstrated no significant difference among different MCPyV detection methods (univariate P=0.954 and multivariate P=0.532) (**Figure 3A**). The combined multivariate HRs were 0.11 (95% CI:0.01-1.09), 0.69 (95% CI:0.35-1.38) and 0.75 (95% CI:0.50-1.13) for the Asian studies, the US studies and the European studies, respectively. However, it showed no significant difference among different continents (P=0.272) (**Figure 3B**). Moreover, there is significant heterogeneity among different countries (P=0.02) (**Figure 3C**).

### Prognostic value of MCPyV positivity for MSS in MCC

**Figure 4:**
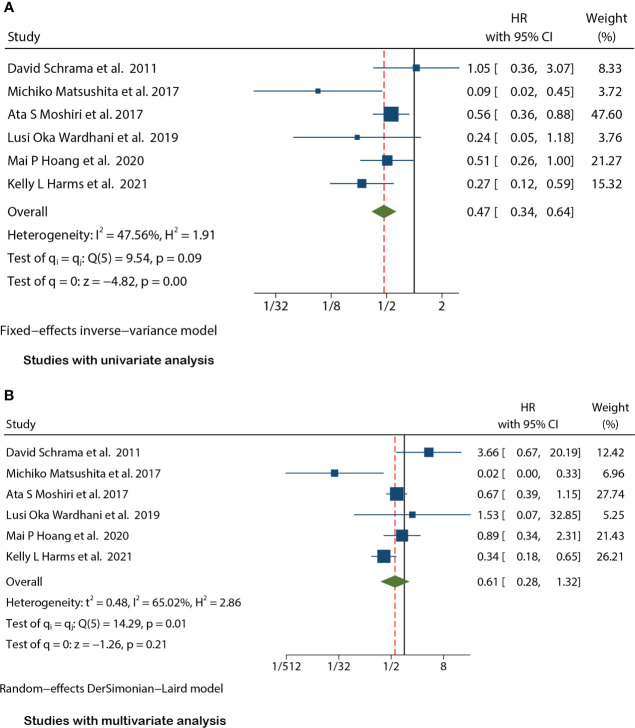
Forest plot of the hazard ratio for the association between the MCPyV positivity and MCC-specific Survival(MSS*)* in patients with Merkel cell carcinoma. **(A)** univariate analysis. **(B)** multivariate analysis.

There were seven studies (12, 15, 17–20, 25) that mentioned the data on MSS. Because the heterogeneity test results were different (univariate I^2^ = 47.56%, P=0.09 and multivariate I^2^ = 65.02%, P=0.01), we conducted a univariate meta-analysis with a fixed-effects model and a multivariate meta-analysis with a random-effects model, respectively. The combined univariate HR of the studies assessing MCPyV positivity on MSS was 0.47 (95% CI:0.34-0.64, P=0.000), indicating that MCPyV positivity may predict better MSS. However, the combined multivariate HR result was 0.61 (95% CI:0.28-1.32, P=0.209), indicating that there was no significant correlation between MCPyV positivity and MSS (**Figure 4**).

### Prognostic value of MCPyV positivity for RFS in MCC

**Figure 5:**
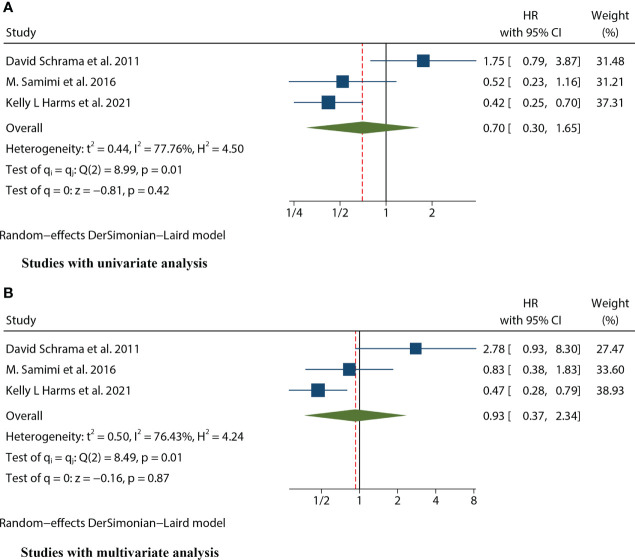
Forest plot of the hazard ratio for the association between the MCPyV positivity and Recurrence-free Survival (RFS) in patients with Merkel cell carcinoma. **(A)** univariate analysis. **(B)** multivariate analysis.

Three studies (12, 16, 25) mentioned the data on RFS. Because of significant heterogeneity in both univariate (I^2^ = 77.76%, P=0.01) and multivariate meta-analysis (I^2 =^ 76.43%, P=0.01), we used the random effects model for both meta-analyses. The combined univariate HR of the studies assessing the impact of MCPyV positivity on RFS was 0.70 (95% CI:0.30-1.65, P=0.421). Meanwhile, the combined multivariate HR was 0.93 (95% CI:0.37-2.34, P=0.873), both indicating no significant correlation between MCPyV positivity and RFS (**Figure 5**).

### Prognostic value of MCPyV positivity for PFS in MCC

**Figure 6:**
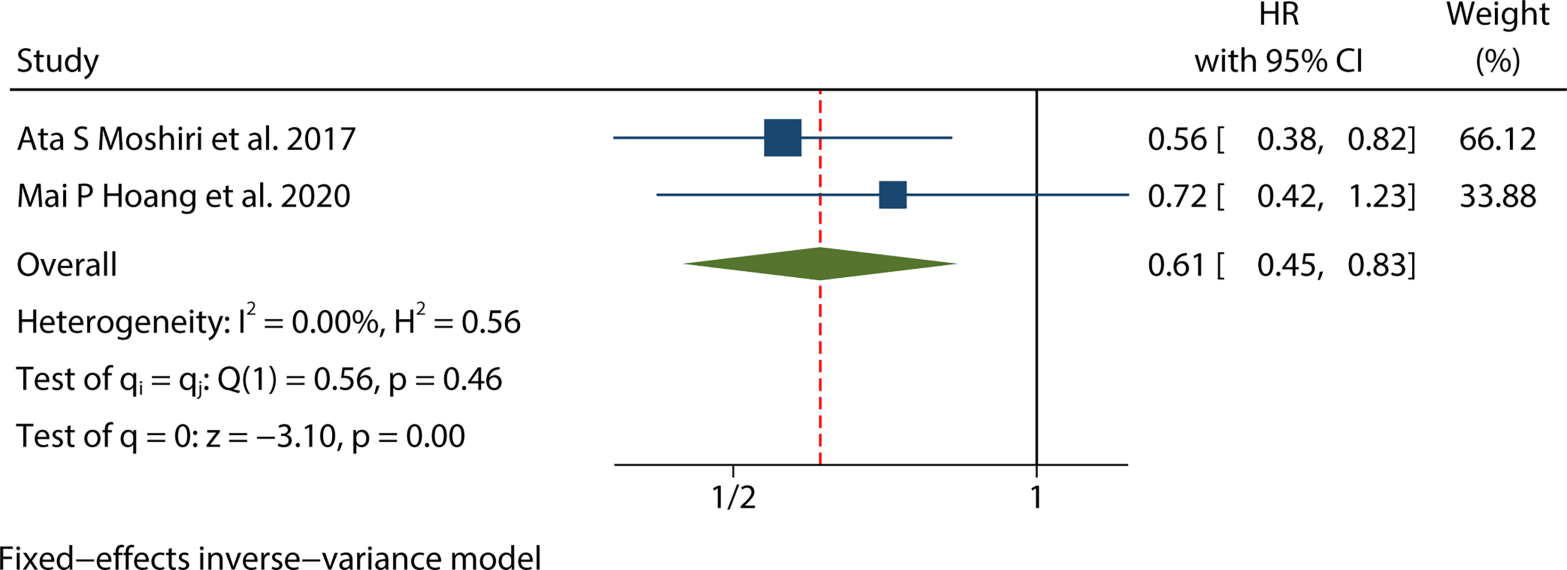
Forest plot of the hazard ratio for the association between the MCPyV positivity and Progression-free Survival (PFS) in patients with Merkel cell carcinoma. (univariate analysis).

Two studies (18, 20) presented the univariate analysis data on PFS, while only one of them mentioned the multivariate analysis data. Since there was no significant heterogeneity (I^2^ = 0.00%, P=0.46), this univariate meta-analysis was conducted using a fixed effects model. The pooled univariate HR of the studies assessing the impact of MCPyV positivity on PFS was 0.61 (95% CI:0.45-0.83, P=0.002), indicating that MCPyV positivity was an indicator of a good prognosis for MCC (**Figure 6**). However, we could not combine the multivariate HRs because one of the studies was lack of the data.

### MCPyV positivity and clinicopathological characteristics in MCC

The clinicopathological characteristics of patients with MCPyV positivity MCC were described in 8 studies (12, 15, 17–20, 23, 25) including gender, histopathological stage, immunosuppression, and primary site are shown in **Table 4**. The meta-analysis was calculated based on the studies in **Table 4**. We observed that the MCPyV positivity was associated with gender (male vs. female, OR=0.606, 95%CI:0.449–0.817, P=0.001), histopathological stage(AJCC I-II vs. III-IV, OR=1.636, 95%CI:1.126–2.378, P=0.010), primary site (head/neck vs. other sites, OR=0.409, 95%CI:0.221–0.757, P=0.004). However, no association was found between MCPyV positivity and immunosuppression (yes vs. no, OR=0.933, 95%CI:0.417–2.088, P=0.867) (**Table 4**).

**Table 4:** Meta-analysis of reported clinicopathological characteristics in the included studies.

Parameters	Number of studies	Odd Ratio (95%CI)	P value	Test for heterogeneity		
				I²(%)	P	Statistic model
Gender (male vs female)	8 (12, 15, 17–20, 23, 25)	0.606(0.449 ~ 0.817)	0.001	20.86	0.264	fixed
Histopathological stage (I-II vs III-IV)	6 (12, 15, 17–19, 25)	1.636(1.126 ~ 2.378)	0.01	0	0.469	fixed
Immunosuppression (yes vs no)	3 (12, 18, 20)	0.933(0.417 ~ 2.088)	0.867	0	0.419	fixed
Localization(Head/neck vs other sites)	4 (12, 18, 20, 25)	0.409(0.221 ~ 0.757)	0.004	69.48	0.02	random

### Sensitivity analysis

**Figure 7:**
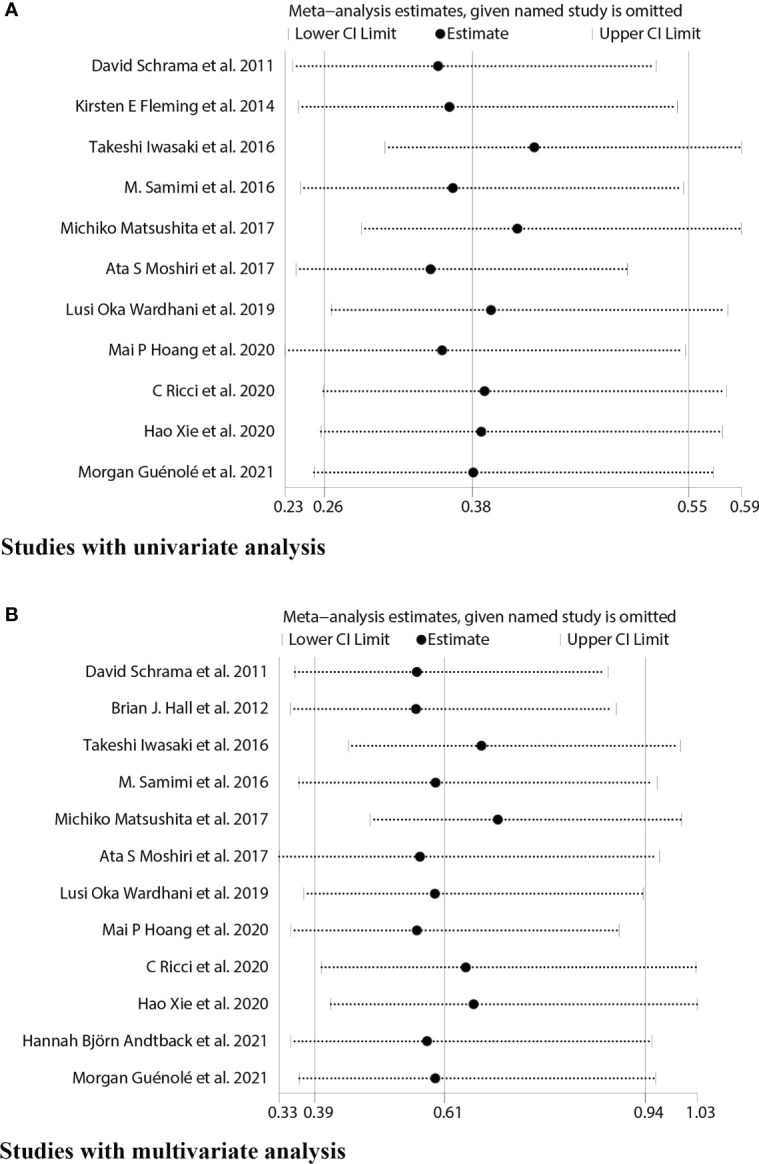
Sensitivity analyses for studies on the association between MCPyV positivity and overall survival(OS). **(A)** univariate analysis. **(B)** multivariate analysis.

We used sensitivity analysis to investigate potential heterogeneity in eligible studies about OS univariate and multivariate analysis, and each article was excluded individually to determine the stability of the combined results (**Figure 7**).

**Figure 8:**
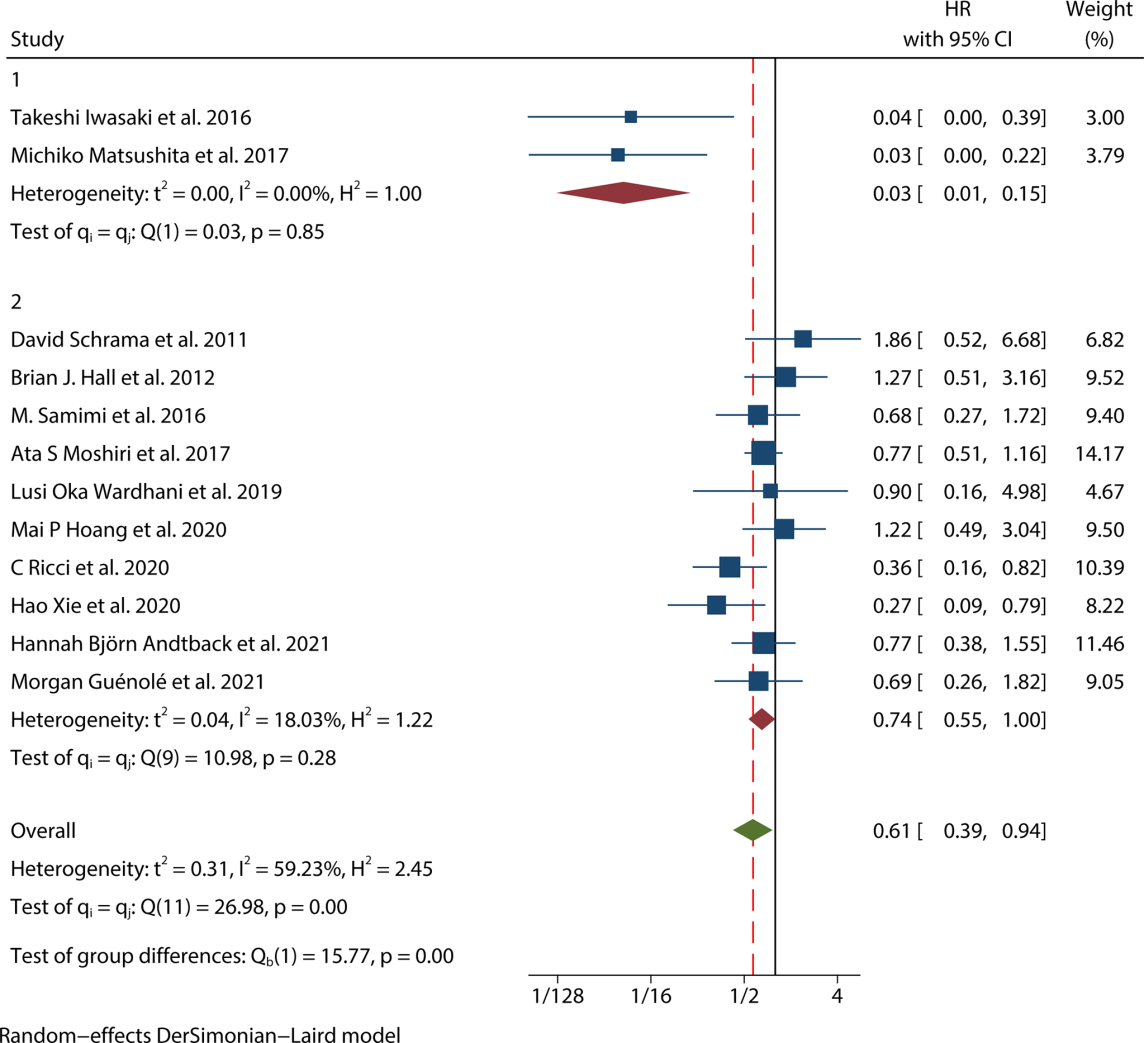
Sensitivity analysis of the association between MCPyV positivity and overall survival.

It showed that the results of the OS univariate meta-analysis did not differ significantly after removing any one of these papers (**Figure 7A**). However, the results of the OS multivariate meta-analysis showed significant heterogeneity after such removal (**Figure 7B**). After excluding two Japanese small sample studies (15, 17), no significant heterogeneity was found in the test of heterogeneity for OS analysis (I^2^ = 18.03%, P=0.28). Furthermore, the new pooled HR for the multivariate analysis of OS in MCPyV-positive versus negative patients was 0.74(95% CI: 0.55–1.00, P=0.047), indicating a good prognostic role of MCPyV positivity (**Figure 8**). Thus, we need to be cautious in concluding the relationship between MCPyV positivity and OS.

### Publication bias

**Figure 9:**
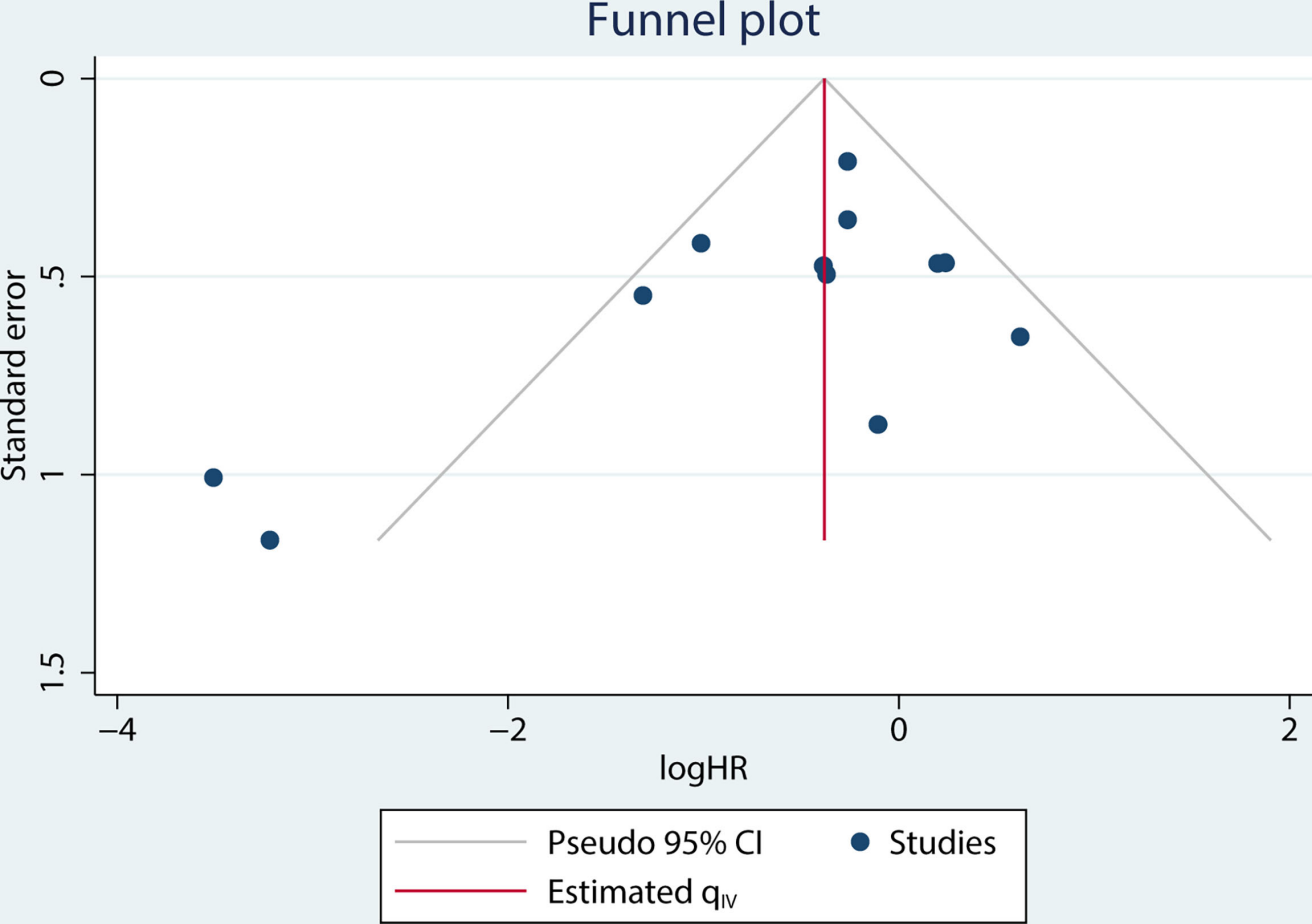
Funnel plot for studies on the association between MCPyV positivity and overall survival (OS).

**Figure 10:**
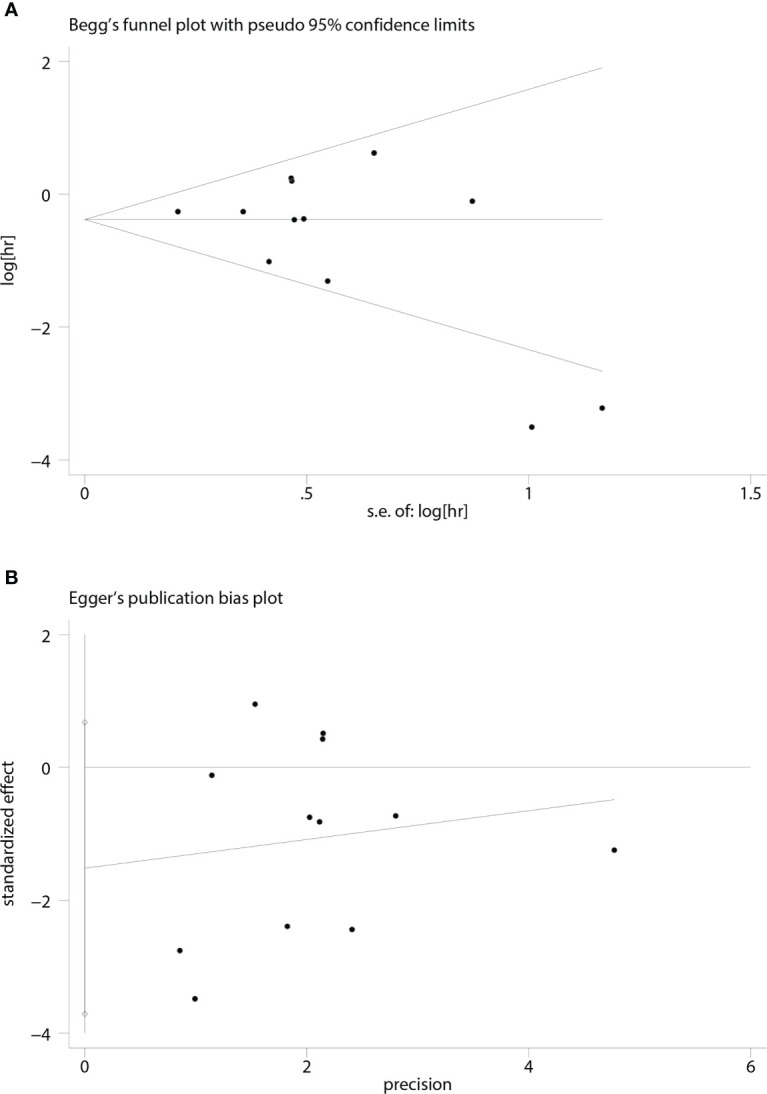
Funnel plots for detecting publication bias in terms of overall survival data. **(A)** Begg’s funnel plot using data of overall survival to detect publication bias; **(B)** Egger’s funnel plot using data of overall survival to detect publication bias.

A funnel plot of the OS multivariate analysis is shown in **Figure 9**, where each point represents an independent study. Moreover, no publication bias was found in the funnel plots used to detect OS data among the articles (Egger’s test, P =0.154; Begg’s test, P = 0.150) (**Figure 10**).

## Discussion

MCPyV is a naked dual-stranded DNA virus of the family Polyomaviridae that has been implicated in the development of MCC (9, 26). MCPyV can be detected in most healthy humans and is known to be persistent in the microbiome *in vivo* without causing any symptoms. Most MCPyV carriers are asymptomatic, and only a small percentage develop MCC (9, 27, 28). The early coding region, the late coding region, and the non-coding regulatory region together constitute the genome of MCPyV. Among them, the early coding region includes two oncoproteins, large T (LT) and small T antigen (ST), which are expressed upon cellular entry and are essential for MCC development and cell survival. The late coding region includes the major capsid proteins VP1, which acts on cellular binding and entry, and the small capsid proteins VP2, which increases infectivity, as well as VP3, whose role is currently unclear (9, 26, 28).

Two rare mutations in MCPyV cause MCC: one mutation gives the virus the ability to clonal integration, and one mutation causes LT antigen to become a truncated form (9, 28). These two mutations are relatively rare, which explains MCC’s rarity despite the MCPyV infection’s commonness. Furthermore, despite the widespread presence of MCPyV VP1 antibodies in the population, LT and ST antibodies can only be detected in less than 1% of healthy subjects (28, 29). In contrast, LT and ST antibodies are detectable in the vast majority of MCC patients, which further suggests that mutations in MCPyV are necessary for carcinogenesis (30). The study also found that patients with MCC who detected high levels of antibodies to MCPyV had better clinical outcomes (31, 32). However, the pathogenic mechanism of MCPyV is still not fully elucidated. In the future, further studies on MCPyV may help to adjust the treatment protocols and diagnostic tools for MCC (33).

Most studies suggest that MCC may have a majority (80%) MCPyV-positive subtype and another minority (20%) MCPyV-negative subtype (27). In addition to MCPyV infection, UV exposure is a significant risk factor for MCC, and it has the potential to trigger genetic mutations, which would lead to immunosuppression (2, 9, 28, 34–36). Enrichment of UV-induced mutations detected in most MCPyV-negative MCC and not identified in MCPyV-positive MCC (28, 34–36). Thus, the possibility of MCPyV-negative MCC deriving from UV-driven pathways is high. In addition to having higher UV signatures mutational loads, MCPyV-negative MCC had more frequent TP53 and RB mutations, higher JAG1 expression, and was also involved in activation of the JAK-STAT and MEK-ERK pathways compared to MCPyV-positive MCC (37–40). These studies may partly explain why MCPyV-positive patients have a better prognosis than virus-negative patients.

It is now believed that Merkel cells are no longer the origin of MCC. Moreover, MCC may originate from cells of two different germ layers: MCPyV-positive MCC from fibroblasts of the mesoderm and MCPyV-negative MCC from keratin-forming cells of the ectoderm (41–43). The original cells of McPyv-positive MCC are thought to be pro-B lymphocytes or pre-B lymphocytes, dermal fibroblasts, or epidermal precursor cells (41–44). One of the characteristics of pro- and pre-B lymphocytes is the expression of immunoglobulins (Igs). Since Igs are expressed in MCCs, this suggests that B lymphocytes may be their cellular ancestors. At least one of Igs was found to be expressed in MCPyV-positive MCCs (IgG, IgA, IgM, or Igκ), but not in MCPyV-negative MCCs (45). In contrast, the original cells of virus-negative MCC may be keratinocytes/epidermal precursor cells that have been severely UV-mutated, which is characteristic of epidermal-derived cancers, such as squamous cell carcinoma (SCC) and melanoma (41, 42). MCPyV-negative MCC cases reported positivity for CK20, synaptophysin, and EMA in combination with a SCC in situ, which was not found in MCPyV-positive MCC (46). In summary, the different genetic mutations and original cells of the two subtypes of MCC may result in different prognoses, which need to be further explored.

The debate on the potential value of MCPyV positivity on the prognosis of MCC patients remains inconclusive. Our study was designed to clarify this issue. Our meta-analysis ultimately included a total of 14 eligible articles involving 1595 patients. After analysis, it was found that MCPyV positivity may be an indicator of a favorable prognosis for OS/PFS in MCC patients, without a significant association with MSS/RFS. Thus, our meta-analysis supports the hypothesis that MCPyV is an indicator of favorable prognosis in MCC patients. Furthermore, our subgroup analyses between different detection methods and between different continents did not reveal significant heterogeneity. As for clinicopathologic factors, MCPyV positivity was associated with gender, histopathological stage, and primary site, while there was no significant correlation between it and immunosuppression. To our best knowledge, our study is the first to provide the most comprehensive and up-to-date systematic review and meta-analysis specifically addressing the relationship between MCPyV positivity and prognosis in MCC patients.

Given that our meta-analysis may have some limitations and shortcomings, the results should be interpreted with caution. First, because all the studies we included were published in English, publication bias in different languages may exist. Second, we used different methods to extract and transform HR values and their 95% CIs from different papers, which may cause imperceptible errors caused by using different methods, but this is unavoidable when collecting data. Third, this meta-analysis relies on observation-based data, as neither are randomized trials available at present nor are they likely to be carried out in the future. Therefore, biases that cannot be measured in individual observational studies must be considered. Fourth, sensitivity analysis showed that the conclusion of the relationship between MCPyV positivity and OS was unstable, possibly due to the small sample sizes of the two Japanese studies. Therefore, based on the above points, it is necessary to be cautious when drawing conclusions about the prognostic value of MCPyV positivity for MCC patients.

## Conclusion

In conclusion, the meta-analysis of this study demonstrated that MCPyV-positive MCC patients had a better survival rate than MCPyV-negative patients, both in terms of OS and PFS rates. Meanwhile, this meta-analysis suggested that MCPyV positivity may predict female gender, earlier histopathological stage, and better primary site of MCC. In addition, more high-quality and multicenter studies should be conducted further to elucidate the impact of MCPyV positivity on MCC patients.

## Data availability statement

The original contributions presented in the study are included in the article/supplementary material. Further inquiries can be directed to the corresponding authors.

## Author contributions

AY: conceptualization, methodology, visualization; writing—original draft preparation. YC, JC, and LY: supervision, funding acquisition. AY, WW, and LY: methodology, visualization. WW, LY, and YH: data curation, sample contribution. AY: conceptualization, formal analysis, investigation, writing— review and editing, supervision. All authors have read and agreed to the published version of the manuscript.

## Funding

This research article was funded by the Science and Technology Support Program of Science and Technology Department of Sichuan Province (2020YFS0267), the Natural Science Foundation of Sichuan Province (2022NSFSC0717), the National Natural Science Foundation of China (81871574).

## Conflict of interest

The authors declare that the research was conducted in the absence of any commercial or financial relationships that could be construed as a potential conflict of interest.

## Publisher’s note

All claims expressed in this article are solely those of the authors and do not necessarily represent those of their affiliated organizations, or those of the publisher, the editors and the reviewers. Any product that may be evaluated in this article, or claim that may be made by its manufacturer, is not guaranteed or endorsed by the publisher.
